# The bi-phasic behaviour of grey matter networks after the first demyelinating attack

**DOI:** 10.1093/braincomms/fcaf367

**Published:** 2025-09-23

**Authors:** Sara Collorone, Giuseppe Pontillo, Michael A Foster, Ferran Prados, Baris Kanber, Marios C Yiannakas, Ailbhe Burke, Lola Ogunbowale, Indran Davagnanam, Claudia A M Gandini Wheeler-Kingshott, Frederik Barkhof, Olga Ciccarelli, Ahmed T Toosy

**Affiliations:** NMR Research Unit, Queen Square MS Centre, Department of Neuroinflammation, UCL Institute of Neurology, Faculty of Brain Sciences, University College London, London WC1B 5EH, UK; NMR Research Unit, Queen Square MS Centre, Department of Neuroinflammation, UCL Institute of Neurology, Faculty of Brain Sciences, University College London, London WC1B 5EH, UK; Department of Radiology and Nuclear Medicine, Amsterdam University Medical Centers, Vrije Universiteit, Amsterdam 1081 BT, Netherlands; Department of Advanced Biomedical Sciences, University ‘Federico II’, Naples 80138, Italy; NMR Research Unit, Queen Square MS Centre, Department of Neuroinflammation, UCL Institute of Neurology, Faculty of Brain Sciences, University College London, London WC1B 5EH, UK; NMR Research Unit, Queen Square MS Centre, Department of Neuroinflammation, UCL Institute of Neurology, Faculty of Brain Sciences, University College London, London WC1B 5EH, UK; Centre for Medical Image Computing, Department of Medical Physics and Biomedical Engineering, University College London, London WC1B 6BT, UK; eHealth Center, Universitat Oberta de Catalunya, Barcelona 08018, Spain; NMR Research Unit, Queen Square MS Centre, Department of Neuroinflammation, UCL Institute of Neurology, Faculty of Brain Sciences, University College London, London WC1B 5EH, UK; Centre for Medical Image Computing, Department of Medical Physics and Biomedical Engineering, University College London, London WC1B 6BT, UK; NMR Research Unit, Queen Square MS Centre, Department of Neuroinflammation, UCL Institute of Neurology, Faculty of Brain Sciences, University College London, London WC1B 5EH, UK; Moorfields Eye Hospital, London EC1V 2PD, UK; Moorfields Eye Hospital, London EC1V 2PD, UK; Department of Brain Repair and Rehabilitation, University College London Institute of Neurology, Faculty of Brain Sciences, UCL, London WC1N 3AZ, United Kingdom; NMR Research Unit, Queen Square MS Centre, Department of Neuroinflammation, UCL Institute of Neurology, Faculty of Brain Sciences, University College London, London WC1B 5EH, UK; Department of Brain and Behavioural Sciences, University of Pavia, Pavia 27100, Italy; Brain MRI 3T Research Centre, IRCCS Mondino Foundation, Pavia 27100, Italy; NMR Research Unit, Queen Square MS Centre, Department of Neuroinflammation, UCL Institute of Neurology, Faculty of Brain Sciences, University College London, London WC1B 5EH, UK; Department of Radiology and Nuclear Medicine, Amsterdam University Medical Centers, Vrije Universiteit, Amsterdam 1081 BT, Netherlands; Department of Brain Repair and Rehabilitation, University College London Institute of Neurology, Faculty of Brain Sciences, UCL, London WC1N 3AZ, United Kingdom; NMR Research Unit, Queen Square MS Centre, Department of Neuroinflammation, UCL Institute of Neurology, Faculty of Brain Sciences, University College London, London WC1B 5EH, UK; National Institute for Health and Care Research (NIHR) University College London Hospitals (UCLH) Biomedical Research Centre, London W1T 7HA, UK; NMR Research Unit, Queen Square MS Centre, Department of Neuroinflammation, UCL Institute of Neurology, Faculty of Brain Sciences, University College London, London WC1B 5EH, UK

**Keywords:** relapsing–remitting multiple sclerosis, regional radiomics similarity networks, graph theory, longitudinal study, small-world

## Abstract

Multiple sclerosis can be considered a network disease. Accumulating evidence recognizes the following importance of grey matter networks: they only require high-resolution anatomical scans for their extraction, they capture changes beyond detectable atrophy and their alteration is associated with disability progression and cognitive impairment. Therefore, it is crucial to understand their behaviours over the initial years of the disease. This observational longitudinal study aimed to investigate changes in grey matter networks after the first demyelinating attack, and how they correlate with brain damage, disability, and conversion to multiple sclerosis over 3–5 years. So far, in multiple sclerosis, network construction has only been based on cortical grey matter, neglecting a possible role for deep grey matter. We applied a radiomics-based network methodology incorporating both deep and cortical grey matter. Patients recruited within 3 months of disease onset and healthy controls attended study visits at 6 months, 1 year, 3 years and 5 years. Study visits included physical and cognitive scales and brain MRI scans. Individual grey matter networks were constructed by computing the correlations between T1w-based radiomic features extracted from any pair of regions of the Brainnetome atlas and characterized with measures of network integration (global efficiency and characteristic path length), segregation (clustering coefficient and modularity), resilience (assortativity) and smallworldness. Additionally, eigenvector centrality was computed for all brain regions as a measure of nodal influence. We enrolled 89 patients (median follow-up 7 months, range 0–75) and 31 healthy controls. Patients showed higher global efficiency, lower shortest characteristic path length and higher smallworldness than controls suggesting a reorganization that prioritize more efficient global communication over local processing. Over time, patients’ networks converged towards healthy controls’ values by increasing the shortest characteristic path length and decreasing the smallworldness. Assortativity, and the eigenvector centrality in the right ventromedial putamen decreased compared with controls. All the observed changes were driven by non-converters to multiple sclerosis. This study shows that grey matter networks adopt a biphasic behaviour. They respond to the demyelinating event with an increase in nodal integration and then converge to healthy control values. In the process, however, their network resilience is compromised. This suggests that a single demyelinating event has longer-lasting effects on grey matter networks, even in non-converters, and that studying these networks may reveal relevant changes that are not captured by conventional MRI in the early years of the disease.

## Teaser

Collorone and Pontillo *et al*. described a biphasic behaviour of grey matter networks after the first demyelinating attack, with initial integration boost followed by convergence to healthy controls values in those not converting to multiple sclerosis. The resilience of the network is impaired, suggesting persistent changes undetected by conventional MRI.

## Graphical Abstract

**Graphical Abstract:**
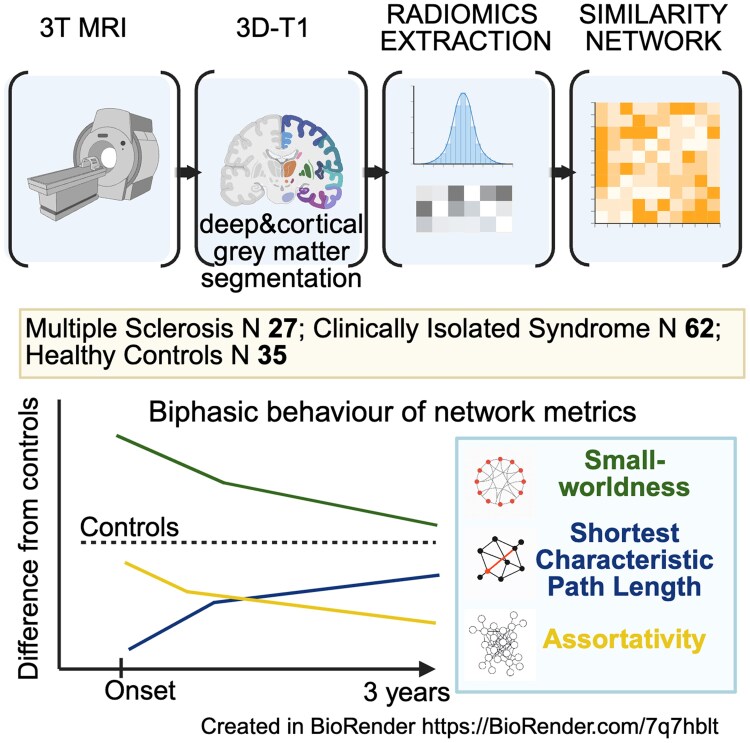

## Introduction

Multiple sclerosis is a chronic disease with CNS damage characterized by inflammation, demyelination and neurodegeneration.^1^ Advances in MRI have improved our understanding of these processes *in vivo,*^2^ but MRI measures of CNS damage still provide an incomplete view.^3^ Multiple sclerosis is also a network disease: the brain’s capacity to compensate for structural damage through functional reorganization diminishes over time and this can contribute to disability.^4^

Many studies have now characterized grey matter networks in multiple sclerosis, both at group^5-9^ and individual level.^10-14^ These network analyses offer valid biological substrates^15^ and clinical relevance as they correlate with progression^11^ and cognitive decline,^13,14^ even when adjusting for grey matter atrophy. However, only two studies have examined the behaviour of grey matter networks at the onset of multiple sclerosis, one possessed a cross-sectional design^12^ and the other used a group-level analysis.^6^ The early stages of multiple sclerosis can provide crucial insights into its pathogenesis and the development and progression of the disease. It is therefore of particular interest to understand how grey matter networks evolve in the early stages of relapsing–remitting multiple sclerosis and clinically isolated syndrome (CIS). Additionally, current literature is only based on cortical grey matter networks, neglecting a possible role for deep grey matter. The deep grey matter appears to play an important role in the progression of the disease^16-18^ and alterations in its structures may be present from the beginning and proceed throughout the disease.^19^

In this study, we consecutively recruited patients after their first demyelinating event who were followed up over up to 5 years. We extracted single-subject grey matter networks from both deep and cortical grey matter using a radiomics-based methodology,^20^ which can evaluate more complex features compared with conventional volumetric analyses.^21^

Our aim was to understand how grey matter networks evolve during the first years of the disease and if changes are related to brain damage and disability accrual.

In recent decades, there has been an effort to redefine the phenotypes of multiple sclerosis using MRI abnormalities which reflect pathogenetic mechanisms.^17^ Therefore, our work also assessed whether changes in grey matter networks distinguished between people who converted to multiple sclerosis because of new clinical and/or MRI activity, and people who did not (i.e. CIS).

## Materials and methods

### Standard protocol approvals, registrations and patient consents

This study was conducted at University College London (UCL), Queen Square Institute of Neurology (UCL ethical committee approval: 13/LO/1762; 13/0231-CIS2013). All subjects gave written informed consent.

### Participants

We prospectively recruited patients at the onset of their first demyelinating episode from the National Hospital of Neurology and Neurosurgery and Moorfields Eye Hospital in London, United Kingdom. Inclusion criteria were assessment within 3 months of symptom onset; age between 18 and 65 years; and ability to provide written informed consent in English and undergo MRI. Exclusion criteria included known neurological disease (other than CIS or multiple sclerosis); the presence of antibodies against aquaporin-4 or myelin oligodendrocyte glycoprotein, routinely assessed in patients with optic neuritis or myelitis; pregnancy or breastfeeding; and the presence of magnetically sensitive or otherwise MRI-incompatible implants. We also recruited age and sex-matched healthy controls.

**Figure 1:**
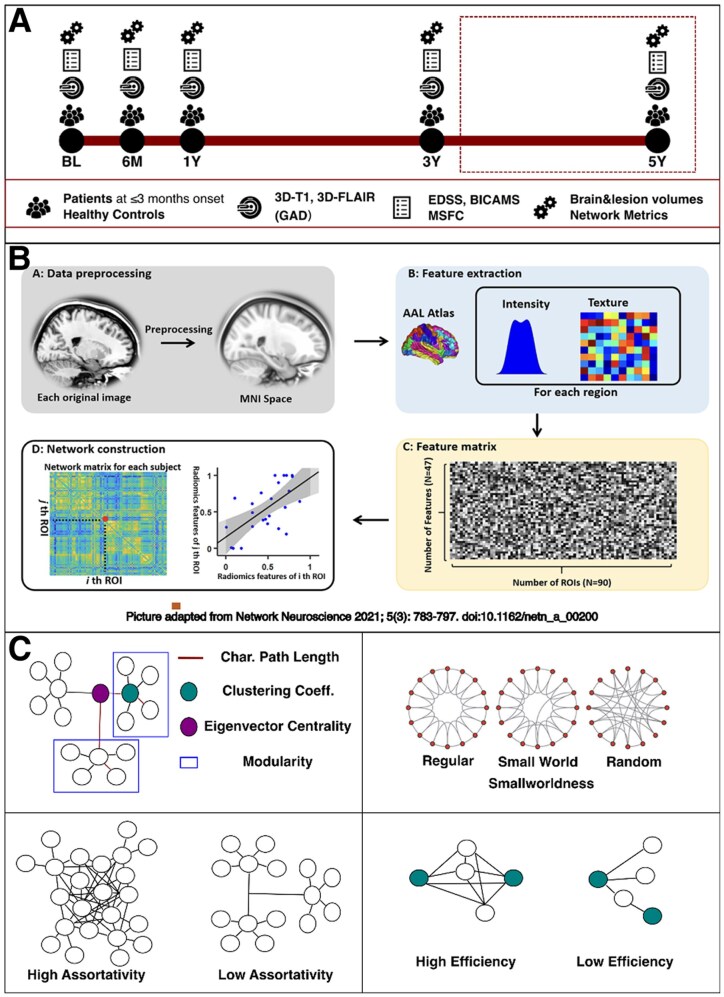
**Study methods.** (**A**) Study plan; (**B**) network extraction; (**C**) network metrics. Network extraction is adapted from Zhao *et al*. Regional radiomics similarity networks (R2SNs) in the human brain: reproducibility, small-world properties and a biological basis. *Network Neuroscience*. 2021;5(3):783. doi:10.1162/NETN_A_00200.^20^ AAL, Automated Anatomical Labelling; BL, baseline; BICAMS, Brief International Cognitive Assessment for Multiple Sclerosis; Char., characteristic; Coeff., coefficient; EDSS, expanded Disability Status Scale; FLAIR, FLuid-Attenuated Inversion Recovery; GAD, gadolinium; M, months; MNI, Montreal Neurological Institute; MSFC, Multiple Sclerosis Functional Composite; ROI, region of interest; Y, year(s).

All participants underwent the same MRI protocol at study entry, 6 months, 1 year, 3 years and 5 years. Additionally, patients had a comprehensive clinical assessment, detailed below, at each time point (Fig. 1). Multiple sclerosis was diagnosed based on the 2017 revision of the McDonald criteria. At baseline, patients had a lumbar puncture if clinically indicated. Individuals who did not meet the 2017 revision of the McDonald criteria during the course of the study were defined as CIS.

### MRI protocol

We used a 3T Achieva MRI scanner (Philips Medical Systems, Best, Netherlands), upgraded to a 3T Philips Ingenia CX during the study, with a 32-channel head coil. All participants underwent structural MRI of the brain at each visit including the following sequences: axial proton density (PD)/T2-weighted imaging; 3D T1-weighted magnetization-prepared turbo field echo; and 3D fluid-attenuated inversion recovery (FLAIR).

For clinical purposes, all subjects at each time point also underwent spinal cord MRI including sagittal PD- and T2-weighted imaging. Patients also had pre- and post-gadolinium sequences of brain and spinal cord. Sequences characteristics are summarized in Supplementary Table 1.

### MRI processing

For all patients, white matter lesions were automatically segmented on 3D FLAIR and 3D T1w scans using SAMSEG,^22^ while FreeSurfer 7.2^23^ was used to obtain estimated total intracranial volume (eTIV)^24^ and gray matter volumes. Thalamic volume was also calculated and then normalized for eTIV.

### Network construction and metric extraction

To obtain single-subject grey matter networks, we used the regional radiomics similarity networks (R2SNs) approach.^20^ Briefly, each T1-weighted volume was nonlinearly registered to the Montreal Neurological Institute (MNI) space using the SyN method implemented in Advanced Normalization Tools (ANTs),^25^ and resampled to 1 mm^3^ voxel resolution. Then, a series of radiomics features (*N* = 47)^26^ were extracted for 246 regions defined by the Brainnetome Atlas.^27^ The parcellation scheme is a fundamental element in the construction of connectivity matrices, as it defines the nodes of the network and consequently shapes the calculation and interpretation of brain connectivity patterns.^28^ The Brainnetome Atlas was utilized as it provides a fine-grained sampling of both cortical and subcortical regions derived from information on both anatomical and functional connections, and for consistency and reproducibility with previous works by Zhao and colleagues.^20,29^ After feature scaling and redundancy elimination, we obtained individual 246 × 246 radiomics similarity matrices where each node represents a region of the Brainnetome Atlas, and edges are computed as the Pearson’s correlation coefficient between interregional radiomics features (Fig. 1B). To obtain a comprehensive characterization of the topological properties of the obtained grey matter networks, we used the Brain Connectivity Toolbox (https://sites.google.com/site/bctnet/) to extract global measures of network integration (global efficiency, characteristic path length), segregation (clustering coefficient, modularity) and resilience (assortativity).^30^ Additionally, smallworldness was computed as the ratio between the normalized clustering coefficient and the normalized characteristic path length,^31^ and eigenvector centrality was extracted for all brain regions as a measure of nodal influence (Fig. 1C).^30^

### Clinical tests

All subjects at all time points completed the following tests: the Expanded Disability Status Scale (EDSS)^32^; the Multiple Sclerosis Functional Composite (MSFC)^33^ including the Timed 25-Foot Walk (T25-FW), 9-Hole Peg Test (9-HPT) and Paced Auditory Serial Addition Test (PASAT); and the Brief International Cognitive Assessment for Multiple Sclerosis (BICAMS)^34^ including Symbol Digit Modality Test (SDMT), Brief Visuospatial Memory Test-Revised (BVMT-R) and California Verbal Learning Test second edition (CVLT-II). We used raw scores for our analysis.

### Statistical analysis

We performed descriptive and statistical analysis using Stata/SE 15.1 (Stata Corporation, College Station, TX, USA).

#### Baseline analysis

We used multivariable linear regression with robust standard errors to assess differences between patients and controls in brain volumes and network metrics with age at baseline and sex as covariates. We also assessed steroids effects on baseline analysis. If significant differences were found, we tested the dependence of the altered network metrics on brain volumes (if altered) and lesion volume by adding these variables as predictors in the model. We conducted a sub-group analysis comparing brain volumes and network metrics between CIS, McDonald 2017 multiple sclerosis and controls. Finally, we assessed the effect of altered network metrics and brain volumes on disability scores.

#### Longitudinal analysis

We used multilevel mixed-effects models to determine the effect of *group* (patients versus controls), age, sex, time (expressed as months from the baseline assessment) and group–time interaction on brain volumes and network metrics over time. Random effects were patient- and visit-specific intercepts and slopes to account for individual variability. We used an unstructured covariance between residuals for repeated measurements of the same individual. If a significant *group* effect was found, we assessed the effect of brain volumes (if altered), lesion volume, disease-modifying treatments (binary variable) and relapses on the altered network metrics. We repeated the analysis defining the *group* factor as CIS, McDonald 2017 multiple sclerosis converters, or controls. We assessed if network metric alterations were associated with changes in disability scores over time. Finally, we assessed if alterations in network metrics at baseline could predict disability and brain volume changes over time.

Our missing data analysis procedures used missing at random (MAR) assumptions. We included patients who were lost at follow up once it was determined that this had happened randomly. We considered as significant *P*-values less than 0.05. For the local regional analyses (i.e. the 246 regions from the Brainnetome atlas), we applied the Benjamini and Hochberg false discovery rate correction for multiple comparisons and we report the q-values.^35^

Initial plans were to analyze network metric changes over a 5-year follow-up. However, our recruitment for the fifth year was affected by the COVID pandemic. For the most considerate use of the data collected, our first analyses used the data only up to 3 years of follow-up. We then separately explored the results at 5 years.

## Results

### Demographics

We recruited 89 patients and 31 controls. No clinically relevant comorbidity was reported. Of these, 37 patients and 6 controls completed 3-year follow-up. Patients and controls did not differ in age and sex. One patient failed the algorithm for network extraction and was discarded from the analysis. Demographic characteristics and clinical features are reported in Table 1.

**Table 1:** Demographics and clinical characteristics

	Patients (*n* = 89)		Controls (*n* = 31)	*P*-value
**Sex** *(female/male)*	59 / 30		17 / 14	>0.05^a^
**Median (range) age at baseline** *(years)*	32 (20–53)		31 (22 ± 49)	>0.05^b^
**CIS subtype** (N)	optic neuritis	73	-	-
	brainstem/cerebellum	7		
	spinal cord	5		
	hemisphere	4		
**Disease classification at baseline** *(CIS/RRMS %)*	70/30%		-	-
**Steroids at baseline** *(N, %)*	45, 51%			
**6-month follow-up** *(N, CIS/RRMS %)*	61, 38/62%		25, -	-
**12-month follow-up** *(N, CIS/RRMS %)*	62, 35.5/65.5%		18, -	-
**3-year follow-up** *(N, CIS/RRMS %)*	37, 27/73%		6, -	-
**Median (range) EDSS** *(at baseline)*	1 (0–3.5)		-	-
**Median (range) EDSS** *(at 3 years)*	1 (0–3)		-	-
**Mean (± SD) T2 lesion volume (ml)** (*at baseline*)	3.2 ± 3.6		-	-
**DMT at 3-year follow-up** *(no/yes)*	16 / 21		-	-

CIS, clinically isolated syndrome; DMT, disease-modifying treatment; EDSS, Expanded Disability Status Scale; RRMS, relapsing–remitting multiple sclerosis; SD, standard deviation.

^a^ *P*-value derived from Pearson’s chi-square test.

^b^ *P*-value derived from linear regression.

### Baseline

#### Brain and lesion volumes

All patients had lower subcortical grey matter volume than controls (*β* = −2 [*95% confidence interval (CI*) = −4, −0.3], *P* = 0.02). Multiple sclerosis patients also had lower normalized thalamic volumes than controls (Table 2).

**Table 2:** Brain volumes and global network metrics at baseline

	All patients			MS patients			CIS patients		
	β	95% CI	*P*-value^a^	β	95% CI	*P*-value^b^	β	95% CI	*P*-value^b^
**eTIV**	−51	−119, 7	>0.05	−10	−98, 78	>0.05	−73	−144, 1	>0.05
**GM**	−13	−36, 10	>0.05	−15	−43, 13	−15	−12	−37, 12	>0.05
**DGM**	−2	−4, −0.3	**0.02**	−2	−4, −0.4	**0.02**	−2	−4, 0.7	>0.05
**Thalamic vol.** ^c^	−0.00009	−0.0003, 0.0001	>0.05	−0.0003	−0.0006, −0.00005	**0.02**	6×10–6	−0.0002, 0.0002	>0.05
**CGM**	−11	−31, 8	>0.05	−14	−38, 9	>0.05	−10	−30, 11	>0.05
**Global Efficiency**	0.006	0.003, 0.01	**0.001**	0.006	0.001, 0.01	**0.02**	0.006	0.002, 0.01	**0.002**
**Characteristic Path Length**	−1	−2, −0.4	**0.002**	−0.9	−2, 0.03	>0.05	−1	−2, −0.5	**0.002**
**Clustering Coefficient**	−0.002	−0.006, 0.001	>0.05	−0.002	−0.006, 0.003	>0.05	−0.003	−0.007, 0.001	>0.05
**Smallworldness**	0.05	0.02, 0.07	**0.002**	0.03	−0.001, 0.07	>0.05	0.05	0.02, 0.08	**0.002**
**Assortativity**	0.004	−0.01, 0.02	>0.05	−0.004	−0.03, 0.02	>0.05	0.008	−0.009, 0.02	>0.05
**Modularity**	−0.004	−0.009, 0.001	>0.05	−0.003	−0.01, 0.004	>0.05	−0.004	−0.01, 0.001	>0.05

Significant *P*-values are shown in bold. CGM, cortical grey matter; CI, confidence interval; CIS, clinically isolated syndrome; DGM, deep grey matter; eTIV, estimated total intracranial volume; GM, grey matter; MS, multiple sclerosis.

^a^ *P*-value of the group–visit interaction (i.e. patients versus healthy controls) from multilevel mixed-effects models adjusted for age and sex.

^b^ *P*-value of the group–visit interaction (i.e. CIS patients and multiple sclerosis patients versus healthy controls) from multilevel mixed-effects.

^c^Normalized for eTIV.

#### Global network metrics

**Figure 2:**
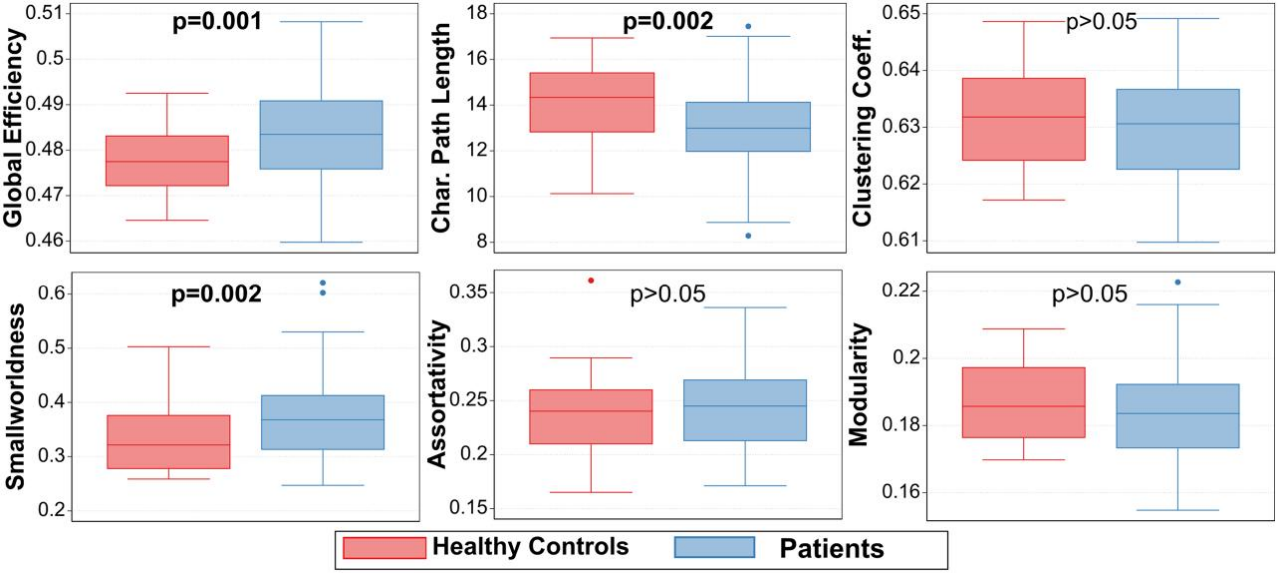
**Boxplots for global network metrics differences between patients and controls at baseline.**  *P*-values are from multivariable linear regression with robust standard errors to assess differences between patients (N 89) and controls (N 31) in network metrics with age at baseline and sex as covariates.

At baseline, patients overall had higher global efficiency (*β* = 0.006 [0.003, 0.01], *P* = 0.001), lower shortest characteristic path length (*β* = −1 [−2, −0.4], *P* = 0.002) and higher smallworldness (*β* = 0.05 [0.02, 0.07], *P* = 0.002) (Table 2; Fig. 2). Subcortical grey matter volume, lesion volume, age and sex were not correlated with these findings. Steroid exposure did not have a significant impact with baseline network metrics. Subgroup analysis showed that while both multiple sclerosis and CIS patients had higher global efficiency than healthy controls, only CIS patients had lower characteristic path length and higher smallworldness than healthy controls (Table 2).

#### Eigenvector centrality

Patients did not differ significantly in eigenvector centrality from controls (*q-value* > 0.05).

#### Disability scores

We did not find any significant relationship between either deep grey matter volume or altered network metrics and disability scores.

### Longitudinal

Out of the 88 patients included in the baseline analysis, 37 were assessed at the three-year follow-up, of whom 27 had converted to multiple sclerosis (73%).

#### Brain and lesion volumes

Over time, we did not observe changes in brain volumes in patients compared with healthy controls (Table 3).

**Table 3:** Brain volumes and global network metrics over 3 years

	All patients			MS patients			CIS patients		
	β	95% CI	*P*-value^a^	β	95% CI	*P*-value^b^	β	95% CI	*P*-value^b^
**eTIV**	0.8	−0.3, 2	>0.05	1	−0.2	>0.05	0.7, 2	−0.6, 2	>0.05
**GM**	−0.03	−0.2, 0.1	>0.05	−0.07	−0.2, 0.2	>0.05	−0.05	−0.3, 0.2	>0.05
**DGM**	−0.01	−0.02, 0.002	>0.05	−0.01	−0.03, 0.003	>0.05	−0.009	−0.02, 0.007	>0.05
**Thalamic vol.** ^c^	−0.002	−0.005, 0.002	>0.05	5.6×10^−6^	−0.00001, 1.5×10^−6^	>0.05	4.6×10^−6^	−0.00001, 2.7×10^−7^	>0.05
**CGM**	−0.005	−0.2, 0.2	>0.05	0.02	−0.2, 0.2	>0.05	−0.03	−0.2, 0.2	>0.05
**Global Efficiency**	−0.00006	−0.0002, 0.00006	>0.05	−0.0001	−0.0002, 0.00002	>0.05	−0.00001	−0.0001, 0.0001	>0.05
**Characteristic Path Length**	0.03	0.006, 0.05	**0.01**	0.02	−0.002, 0.05	>0.05	0.03	0.01, 0.06	**0.01**
**Clustering Coefficient**	0.00006	−0.0001, 0.0002	>0.05	−0.00005	−0.0002, 0.0001	>0.05	0.0002	0.00001, 0.0003	**0.04**
**Smallworldness**	−0.001	−0.002, −0.0001	**0.03**	−0.001	−0.002, 0.0002	>0.05	−0.001	−0.003, −0.0001	**0.03**
**Assortativity**	−0.0004	−0.0007, −0.00003	**0.03**	−0.0004	−0.0008, 0.00004	>0.05	−0.0004	−0.001, −0.00001	**0.04**
**Modularity**	−0.00008	−0.0003, 0.00010	>0.05	−0.0001	−0.0003, 0.00007	>0.05	−0.00002	−0.0002, 0.0002	>0.05

Significative *P*-values are in bold.

Abbreviations: CGM, cortical grey matter; CI, confidence interval; CIS, clinically isolated syndrome; DGM, deep grey matter; eTIV, estimated total intracranial volume; GM, grey matter; MS, multiple sclerosis.

^a^ *P*-value of the group–visit interaction (i.e. patients versus healthy controls) from multilevel mixed-effects models adjusted for age and sex.

^b^ *P*-value of the group–visit interaction (i.e. CIS patients and multiple sclerosis patients versus healthy controls) from multilevel mixed-effects.

^c^Normalized for eTIV.

#### Global network metrics

**Figure 3:**
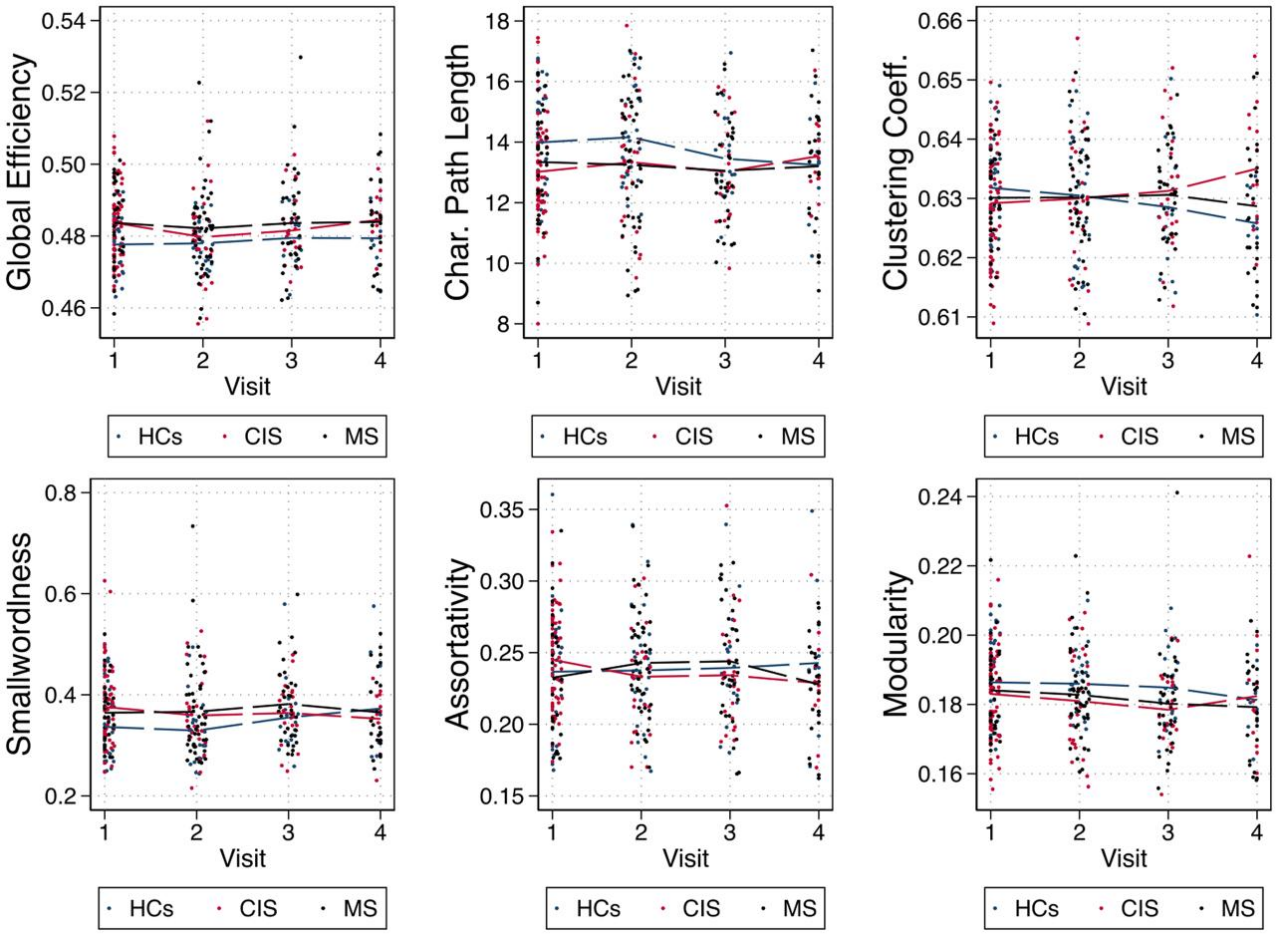
**Global network metrics changes over 3 years in healthy controls, CIS, and MS patients.** Scatter plot of global metric values for each subject at each visit. Dashed lines represent the means for each variable. *P*-values are from multilevel mixed-effects models to determine the effect of *group* (patients versus controls), age, sex, time (expressed as months from the baseline assessment), and group–time interaction on brain volumes and network metrics over time (6 months: CIS N 23, MS N 38, HC N 25; 1 year: CIS N 22, MS N 40, HC N 18; 3 years: CIS N 10, MS N 27 HC N 6). Random effects were patient- and visit-specific intercepts and slopes to account for individual variability. We used an unstructured covariance between residuals for repeated measurements of the same individual. BL, baseline; Char., characteristic; CIS, clinically isolated syndrome; HCs, healthy controls; M, months; MS, multiple sclerosis; Y, year(s).

Over time, in all patients the shortest characteristic path length increased (*β* = 0.03 [0.006, 0.05], *P* = 0.01), and both smallworldness and assortativity decreased (*β* = −0.001 [−0.002, −0.0001], *P* = 0.03; *β* = −0.0004 [−0.0007, −0.00003], *P* = 0.03, respectively) compared to healthy controls. Age, sex, lesion volume, use of disease-modifying treatments and relapses were not correlated to the observed changes. In the subgroup analysis, only the non-converters showed these changes in global network metrics when compared with healthy controls (Table 3, Fig. 3).

#### Eigenvector centrality

Over time, all patients had a greater decrease in eigenvector centrality than healthy controls in the right ventromedial putamen (*β* = − 0.03 [−0.04, −0.01], *P* = 0.00004, *q-value* = 0.01) (see Supplementary Table 2). Age, sex, lesion volume, use of disease-modifying treatments and relapses were not correlated to the observed changes.

#### Disability

Changes in disability were dependent from relapses, and independent from changes in global and local network metrics, and use of disease-modifying treatments. We observed an improvement in the T25-FW speed (*β* = −0.0005[−0.0008, −0.0002], *P* = 0.004) maintained when adding relapses to the model (Table 4).

**Table 4:** Disability scores over 3 years and their relationships with relapse rate

	Not including relapses in the model			Including relapses in the model		
Clinical Test	β	95% CI interval	*P*-value^a^	β	95% CI interval	*P*-value^a^
**EDSS**	0.009	0.0002, 0.02	0.05	0.4	0.2, 0.7	**0.001**
**T25-FW**	−0.0005	−0.0008, −0.0002	**0.004**	−0.008	−0.02, −0.0002	**0.04**
**9-HPT**	0.00001	−0.00008, 0.0001	>0.05	−0.001	−0.003, −0.0002	**0.02**
**PASAT**	−0.004	−0.08, 0.07	>0.05	−4	−6, −2	**0.001**
**SDMT**	0.05	−0.04, 0.1	>0.05	−1	−4, 1	>0.05
**CVLT-II**	0.03	−0.07, 0.07	>0.05	−6	−8, −4	**<0.0001**
**BVMT-R**	−0.005	−0.05, 0.03	>0.05	−3	−4, −1	**<0.0001**

Significative *P*-values are in bold.

**Abbreviations**: BVMT-R, Brief Visuospatial Memory Test-Revised; CI, confidence interval; CVLT-II, California Verbal Learning Test second edition; EDSS, expanded disability status scale; 9-HPT, 9-Hole Peg Test; PASAT, Paced Auditory Serial Addition Test; SDMT, Symbol Digit Modality Test; T25-FW, Timed 25-Foot Walk.

^a^Estimated change of the dependent disability variable over time from multilevel mixed-effects models adjusted for age, sex ± relapses since previous visit.

### Five-year follow-up

Twenty-three patients and six healthy controls completed five-year follow-up. Among patients, nine remained as CIS and 14 had converted to multiple sclerosis. Patients showed a decrease in deep grey matter volume compared to controls (*β* = −0.01 [−0.02, −0.004] *P* = 0.007) that was associated with multiple sclerosis conversions, and not with relapses and use of disease-modifying treatments (Table 5). We observed weak evidence for a decrease in assortativity in patients compared with controls (*β* = −0.0003 [−0.0006, −0.0000007], *P* = 0.06). CIS patients showed an increase in clustering coefficient and characteristic path length, and a decrease in smallworldness (Table 5). There was deterioration in T25-FW performance (*β* = −0.0006 [−0.0008, −0.0003], *P* < 0.0001) independent from relapses, changes in global network metrics and use of disease-modifying treatments.

**Table 5:** Brain volumes and global network metrics over 5 years

	All patients			MS patients			CIS patients		
	β	95% CI	*P*-value^a^	β	95% CI	*P*-value^b^	β	95% CI	*P*-value^b^
**eTIV**	0.3	−0.8, 1	>0.05	0.8	−0.09, 2	0.08	−0.05	−2, 0.9	>0.05
**GM**	−0.01	−0.2, 0.1	>0.05	−0.01	−0.2, 0.2	0.9	−0.003	−0.2, 0.2	>0.05
**DGM**	−0.01	−0.02, −0.0007	**0.04**	−0.02	−0.03, −0.01	**0.003**	−0.01	−0.02, 0.002	>0.05
**Thalamic Vol.** ^c^	−2.4 × 10^−6^	−6.4 × 10^−6^, 1.6 × 10^−6^	>0.05	−4.4 × 10^−6^	−8.6 × 10^−6^, −2.8 × 10^−7^	**0.04**	−1 × 10^−6^	−5.4 × 10^−6^, 3.4 × 10^−6^	>0.05
**CGM**	0.002	−0.1, 0.1	>0.05	0.01	−0.1, 0.2	0.9	−0.0003	−0.2, 0.2	>0.05
**Global Efficiency**	−0.00004	−0.0001, 0.0001	>0.05	−0.00005	−0.0002, 0.0001	0.4	−0.00002	−0.0001, 0.00009	>0.05
**Characteristic Path Length**	0.02	−0.009, 0.04	>0.05	0.005	−0.02, 0.03	0.6	0.03	0.003, 0.05	**0.03**
**Clustering Coefficient**	0.0001	−0.0001, 0.0002	>0.05	−0.00002	−0.0001, 0.0001	0.8	0.0001	0.00002, 0.0003	**0.03**
**Smallworldness**	−0.0008	−0.002, 0.0001	>0.05	−0.0006	−0.002, 0.0004	0.2	−0.001	−0.002, −0.00003	**0.04**
**Assortativity**	−0.0003	−0.0006, 0.00001	0.05	−0.0003	−0.0006, 0.0001	0.1	−0.0003	−0.0007, 0.00006	>0.05
**Modularity**	−0.00003	−0.0002, 0.0001	>0.05	−0.0001	−0.0002 0.0001	0.5	0.00003	−0.0001, 0.0002	>0.05

Significative *P*-values are in bold.

Abbreviations: CGM, cortical grey matter; CI, confidence interval; CIS, clinically isolated syndrome; DGM, deep grey matter; eTIV, estimated total intracranial volume; GM, grey matter; MS, multiple sclerosis.

^a^ *P*-value of the group–visit interaction (i.e. patients versus healthy controls) from multilevel mixed-effects models adjusted for age and sex.

^b^ *P*-value of the group–visit interaction (i.e. CIS patients and multiple sclerosis patients versus healthy controls) from multilevel mixed-effects.

^c^Normalized for eTIV.

## Discussion

By examining grey matter networks at the earliest stage of the disease, we have provided evidence for bi-phasic behaviour. The networks responded to the initial demyelinating attack by increasing nodal integration (i.e. reducing shortest path length and increasing global efficiency). Their topology also deviated from the small-world configuration typical of natural biological systems,^36^ which normally provides balance between short and long-range connections, in favour of a more clustered configuration (i.e. a high smallworldness, typical of regular networks, such as lattices).

Then, over 3 years they began to realign back towards healthy control metrics (i.e. by increasing shortest path length and decreasing smallworldness). The fact that this process of network *re-normalization* has been driven by non-converters has different implications. On the one hand, this means that when there is again inflammatory disease activity (i.e. in converters), grey matter networks respond by shifting to a more integrated configuration, as witnessed after the first attack. On the other hand, this may indicate more efficient repair mechanisms in non-converters, either sustained by remyelinating processes inside the lesion or processes promoting cortical plasticity. These complex dynamics of brain connectivity in early multiple sclerosis call for further research into the mechanisms underpinning these changes.

We also observed reduction in assortativity over time in patients. Assortativity is the tendency for nodes to connect with others that have a similar degree of connectivity. Higher assortativity means that highly connected regions (hubs) prefer to connect with other hubs, while less connected regions connect with each other. So as networks attempt to restore their normal organization, highly connected nodes may lose their preferential links with other hubs with a possible negative impact on the network resilience. This result implies that even a single demyelinating attack can leave longer-lasting changes in the grey matter network structure, even in patients remaining CIS. Hence grey matter networks can detect changes that would not be observable on conventional MRI, thereby offering additional pathobiological insights into clinically early stages.

Interestingly, Tur *et al*.^6^ also reported a bi-phasic behaviour after the first demyelinating attack, but only in people who converted to multiple sclerosis. In addition to the different methodology, the discrepancy in results may be due to the characteristics of their cohort, which at baseline included only CIS patients recruited in the pre-treatment era and used the less sensitive 2010 revision of the McDonald criteria. Nevertheless, this concordance highlights the robustness of our findings and is consistent with the idea that grey matter networks analysis provides complementary information to standard analyses of structural damage.

Other studies have focused on single-subject grey matter networks.^37^ Replicating the results of a previous group-level study,^9^ both our group^12^ and Fleischer *et al*.^10^ showed an increase in the clustering coefficient in patients with CIS and early multiple sclerosis, respectively. An increased clustering coefficient indicates strengthened local information flow in grey matter networks. Since it was not associated with changes in cognitive and physical function, it may suggest a compensatory mechanism in response to the initial attack. It is interesting to note that this finding in CIS goes beyond this specific methodology of network analysis.^38^ Multiple sclerosis patients may not be able to adopt this strategy due to other underlying mechanisms that promote white and grey matter damage.^39^ Furthermore, the confirmation of these results at the five-year follow-up (Table 5, *P* = 0.03), despite the smaller sample size, suggests that these changes in network properties are consistent and endure over time.

This hypothesis is consistent with the observed shift towards a more regular network (i.e. increased smallworldness), which is characterized by nodes that are densely connected to their clique of neighbours. As we have captured morphometric similarity across brain regions, the increase in smallworldness suggests a non-random, regionally coordinated pattern of tissue change, possibly reflecting microstructural remodelling (e.g. synaptic pruning, glial activation) as a system-wide response to the demyelinating insult.

While we also documented this shift in topology in our previous multicentre study,^12^ because of its cross-sectional design, we could not capture the dynamic changes over time. Notably, the analysis of single-subject grey matter networks in advanced multiple sclerosis,^13^ like studies using the same methodology in dementia,^40^ showed that cognitively impaired patients tend towards random network topology. However, unlike the subjects in our cohort, these patients already had evident grey matter atrophy. Thus, we can now confirm that the more clustered organization in CIS and early multiple sclerosis represents an early network response to the inflammatory episode, which then reverts to healthy control values over time, but can become random if substantial neurodegeneration occurs. It is also important to note that most network measures were resistant to change over time, suggesting some stability in the brain's grey matter network structure, even in the presence of disease (Fig. 3).

On the other hand, these compensatory findings may reflect underlying processes that precede the onset of the first clinical event. Such early adaptations could involve the preservation of function despite accumulating damage. These observations raise important questions about the ‘true’ onset of pathological changes and the dynamics of grey matter network changes in early multiple sclerosis. Further studies in high-risk cohorts (i.e. individuals with radiologically isolated syndromes or genetic predispositions) are needed to confirm that these patterns are present before clinical manifestation.

This study progresses the field by incorporating certain methodological advances. For the first time in multiple sclerosis, we have used radiomics to construct our grey matter networks, which might provide a more comprehensive assessment of brain tissue compared with simpler measurements such as cortical volume/thickness, including the characterization of complex microstructural changes. In addition, we have included deep grey matter in the network construction. These structures are crucial for cognitive functions^41^ and play a significant role in multiple sclerosis progression.^42^

In our study, patients exhibited deep grey matter atrophy at onset, but this did not influence the global grey matter network structure and local metrics. However, despite no further changes in deep grey matter volume over the years, we observed decreased eigenvector centrality in the right ventromedial putamen. Eigenvector centrality measures a node's importance based on the number of connections its neighbours make. For instance, in social networks, eigenvector centrality can determine the most influential members by measuring the significance of their contacts.^43^ The deep grey matter is a central part of the high-capacity backbone of the global network structure.^44^ Therefore, the decrease in eigenvector centrality in the putamen may represent hub disconnection suffered by the network. Interestingly, there was a decrease in deep grey matter volume at five-year follow-up, driven, as expected, by multiple sclerosis patients. Longer follow-up will clarify if the early changes in eigenvector centrality in these structures are related to undergoing neurodegenerative processes.

Our study has limitations. First, we experienced a participant drop-out during follow-up that limited our sample size. However, we used robust multilevel statistical methods that can mitigate against missing data points. Second, damage in both nodal regions (i.e. the grey matter structures) and the connecting white matter tracts may influence grey matter structural network metrics. Our study did not explore the impact of cortical lesions^45^ and alterations in the normal-appearing tissues,^39^ making it impossible to disentangle the two components. However, we did account for conventional measures of brain structural damage. Therefore, our findings still have clinical relevance, particularly with considering brain atrophy and white matter lesion load. Finally, although our study was limited by a relatively small sample size at 5 years, we believe it is valuable to report these results as they contribute to hypothesis-generating research, and they also highlight differences between short-term remodeling and long-term reorganization.

In conclusion, we have demonstrated that grey matter networks show relevant changes during the initial years of the disease after clinical onset, not captured by conventional MRI metrics, encouraging a reappraisal of CIS pathophysiological mechanisms. Initially, after the first demyelinating attack, CIS and multiple sclerosis networks behave similarly, reinforcing the idea that multiple sclerosis is a continuum in which clinical phenotypes share common disease mechanisms^46^; however, the absence of further attacks over time may encourage recovery of network integrity, but at the cost of permanent changes. Nevertheless, the re-normalization of grey matter networks may indicate a successful resolution of inflammation, possibly due to effective repair mechanisms. CIS patients whose grey matter networks revert to a healthy state may have limited disease or effective plasticity, while those who diverge from healthy controls norms may benefit from early treatment. The possible role of grey matter network metrics as putative biomarkers to detect early changes requires future study.

## Supplementary Material

fcaf367_Supplementary_Data

## Data Availability

Anonymized data can be made available to other qualified researchers on reasonable request and with adequate justification. The code to obtain radiomics similarity networks is available at https://github.com/YongLiuLab/R2SN_construction.
