# Supplementary material for: The bi-phasic behaviour of grey matter networks after the first demyelinating attack
Source: Brain Commun. 2025 Sep 23;7(5):fcaf367. doi: 10.1093/braincomms/fcaf367 (PMC12495409; doi:10.1093/braincomms/fcaf367)
Supplement: fcaf367_Supplementary_Data [file fcaf367_supplementary_data.docx]

**Supplementary Material**

| **Supplementary Table 1. MRI protocol** |
| --- |

| Sequences | Slice orientation | Measured voxel size (mm) | TR (ms) | TE (ms) | Inversion time (ms) |
| --- | --- | --- | --- | --- | --- |
| Head | | | | | |
| 3D T1 | Sag | 1.0x1.0x1 | 6.9 | 3.1 | 821 |
| 3D FLAIR | Sag | 1.2x1.2x1.2 | 8000 | 388 | 2400 |
| PD/T2 TSE | Ax | 1.0x1.3x3 | 3500 | 15/85 |  |
| T1 TSE +Gd^a^ | Ax | 1.0x1.3x3 | 625 | 10 |  |
| Spinal Cord | | | | | |
| T1 TSE + Gd^a^ | Sag | 1.0x1.8x3 | 600 | 8 |  |
| PD/T2 TSE | Sag | 1.0x1.8x3 | 3500 | 22/78 |  |

*Abbreviations***:** Ax: axial; FLAIR: fluid-attenuated inversion recovery; Gd: gadolinium; PD: proton density; Sag: sagittal; TE: echo time; TR: repetition time; TSE: turbo spin-echo.

^a^Patients only

**Supplementary Table 2. Eigenvector centrality in local areas**

| **Lobe** | **Area Name** | **Area Number** | **Coeff.** | **95% CI interval** | **p-value^a^** | **q-value^b^** |
| --- | --- | --- | --- | --- | --- | --- |
| **Frontal Lobe** | *A8m, medial area 8* | 1 | -18.94 | -45.57  7.69 | 0.16 | 0.69 |
|  | *A8m, medial area 8* | 2 | 1.48 | -21.05  24.01 | 0.90 | 0.98 |
|  | *A8dl, dorsolateral area 8* | 3 | -25.12 | -50.28  0.04 | 0.05 | 0.56 |
|  | *A8dl, dorsolateral area 8* | 4 | 10.43 | -13.26  34.11 | 0.39 | 0.84 |
|  | *A9l, lateral area 9* | 5 | -0.81 | -29.33  27.71 | 0.96 | 0.99 |
|  | *A9l, lateral area 9* | 6 | -3.14 | -32.23  25.95 | 0.83 | 0.96 |
|  | *A6dl, dorsolateral area 6* | 7 | -10.31 | -38.28  17.65 | 0.47 | 0.88 |
|  | *A6dl, dorsolateral area 6* | 8 | -8.12 | -35.14  18.90 | 0.56 | 0.88 |
|  | *A6m, medial area 6* | 9 | -21.30 | -41.00  -1.60 | 0.03 | 0.47 |
|  | *A6m, medial area 6* | 10 | 23.02 | -2.01  48.05 | 0.07 | 0.55 |
|  | *A9m,medial area 9* | 11 | -21.60 | -49.32  6.12 | 0.13 | 0.62 |
|  | *A9m,medial area 9* | 12 | -9.71 | -39.86  20.44 | 0.53 | 0.90 |
|  | *A10m, medial area 10* | 13 | -2.20 | -20.75  16.36 | 0.82 | 0.96 |
|  | *A10m, medial area 10* | 14 | 4.07 | -21.07  29.21 | 0.75 | 0.93 |
|  | *A9/46d, dorsal area 9/46* | 15 | -0.16 | -19.30  18.98 | 0.99 | 0.99 |
|  | *A9/46d, dorsal area 9/46* | 16 | -11.66 | -35.45  12.13 | 0.34 | 0.79 |
|  | *IFJ, inferior frontal junction* | 17 | 5.06 | -23.92  34.04 | 0.73 | 0.93 |
|  | *IFJ, inferior frontal junction* | 18 | -15.78 | -44.68  13.12 | 0.28 | 0.77 |
|  | *A46, area 46* | 19 | -1.85 | -29.45  25.74 | 0.90 | 0.98 |
|  | *A46, area 46* | 20 | 30.61 | 8.78  52.44 | 0.01 | 0.29 |
|  | *A9/46v, ventral area 9/46* | 21 | 6.70 | -16.59  30.00 | 0.57 | 0.89 |
|  | *A9/46v, ventral area 9/46* | 22 | -5.25 | -30.05  19.54 | 0.68 | 0.89 |
|  | *A8vl, ventrolateral area 8* | 23 | 0.79 | -25.27  26.86 | 0.95 | 0.99 |
|  | *A8vl, ventrolateral area 8* | 24 | -1.65 | -27.08  23.78 | 0.90 | 0.97 |
|  | *A6vl, ventrolateral area 6* | 25 | 2.42 | -29.67  34.52 | 0.88 | 0.97 |
|  | *A6vl, ventrolateral area 6* | 26 | 4.04 | -18.53  26.61 | 0.73 | 0.93 |
|  | *A10l, lateral area10* | 27 | 19.31 | -9.75  48.36 | 0.19 | 0.70 |
|  | *A10l, lateral area10* | 28 | 45.02 | 14.39  75.66 | 0.00 | 0.24 |
|  | *A44d,dorsal area 44* | 29 | -21.01 | -44.63  2.61 | 0.08 | 0.51 |
|  | *A44d,dorsal area 44* | 30 | -6.30 | -27.59  15.00 | 0.56 | 0.88 |
|  | *IFS, inferior frontal sulcus* | 31 | 13.11 | -15.39  41.60 | 0.37 | 0.81 |
|  | *IFS, inferior frontal sulcus* | 32 | -2.66 | -25.55  20.23 | 0.82 | 0.96 |
|  | *A45c, caudal area 45* | 33 | 5.89 | -18.70  30.48 | 0.64 | 0.87 |
|  | *A45c, caudal area 45* | 34 | -23.02 | -47.86  1.83 | 0.07 | 0.57 |
|  | *A45r, rostral area 45* | 35 | -12.24 | -44.61  20.14 | 0.46 | 0.87 |
|  | *A45r, rostral area 45* | 36 | -17.34 | -49.14  14.46 | 0.29 | 0.76 |
|  | *A44op, opercular area 44* | 37 | -3.77 | -30.38  22.85 | 0.78 | 0.94 |
|  | *A44op, opercular area 44* | 38 | 0.42 | -27.25  28.10 | 0.98 | 0.99 |
|  | *A44v, ventral area 44* | 39 | -22.19 | -46.81  2.43 | 0.08 | 0.58 |
|  | *A44v, ventral area 44* | 40 | -7.57 | -28.97  13.82 | 0.49 | 0.90 |
|  | *A14m, medial area 14* | 41 | -8.67 | -41.29  23.94 | 0.60 | 0.86 |
|  | *A14m, medial area 14* | 42 | 10.88 | -20.36  42.12 | 0.49 | 0.90 |
|  | *A12/47o, orbital area 12/47* | 43 | 22.93 | -8.93  54.79 | 0.16 | 0.70 |
|  | *A12/47o, orbital area 12/47* | 44 | 32.58 | -8.34  73.50 | 0.12 | 0.60 |
|  | *A11l, lateral area 11* | 45 | 28.81 | -17.88  75.50 | 0.23 | 0.69 |
|  | *A11l, lateral area 11* | 46 | -5.60 | -26.64  15.43 | 0.60 | 0.87 |
|  | *A11m, medial area 11* | 47 | -20.86 | -52.90  11.17 | 0.20 | 0.71 |
|  | *A11m, medial area 11* | 48 | 19.02 | -14.25  52.30 | 0.26 | 0.77 |
|  | *A13, area 13* | 49 | 10.49 | -9.13  30.10 | 0.29 | 0.76 |
|  | *A13, area 13* | 50 | 19.75 | -7.49  47.00 | 0.16 | 0.69 |
|  | *A12/47l, lateral area 12/47* | 51 | 9.56 | -24.78  43.89 | 0.59 | 0.87 |
|  | *A12/47l, lateral area 12/47* | 52 | 22.41 | -6.42  51.24 | 0.13 | 0.62 |
|  | *A4hf, area 4(head and face region)* | 53 | -1.38 | -27.79  25.03 | 0.92 | 0.99 |
|  | *A4hf, area 4(head and face region)* | 54 | -15.47 | -42.72  11.78 | 0.27 | 0.77 |
|  | *A6cdl, caudal dorsolateral area 6* | 55 | -10.79 | -35.52  13.93 | 0.39 | 0.84 |
|  | *A6cdl, caudal dorsolateral area 6* | 56 | -11.57 | -36.34  13.20 | 0.36 | 0.83 |
|  | *A4ul, area 4(upper limb region)* | 57 | -38.31 | -70.80  -5.82 | 0.02 | 0.34 |
|  | *A4ul, area 4(upper limb region)* | 58 | -12.55 | -41.43  16.33 | 0.39 | 0.84 |
|  | *A4t, area 4(trunk region)* | 59 | -4.76 | -22.69  13.18 | 0.60 | 0.86 |
|  | *A4t, area 4(trunk region)* | 60 | -24.69 | -59.46  10.09 | 0.16 | 0.68 |
|  | *A4tl, area 4(tongue and larynx region)* | 61 | 39.70 | 7.14  72.27 | 0.02 | 0.35 |
|  | *A4tl, area 4(tongue and larynx region)* | 62 | 18.37 | -7.36  44.09 | 0.16 | 0.70 |
|  | *A6cvl, caudal ventrolateral area 6* | 63 | -3.35 | -26.13  19.42 | 0.77 | 0.95 |
|  | *A6cvl, caudal ventrolateral area 6* | 64 | -6.13 | -30.34  18.07 | 0.62 | 0.87 |
|  | *A1/2/3ll, area1/2/3 (lower limb region)* | 65 | 4.18 | -13.86  22.21 | 0.65 | 0.88 |
|  | *A1/2/3ll, area1/2/3 (lower limb region)* | 66 | 7.84 | -12.39  28.08 | 0.45 | 0.87 |
|  | *A4ll, area 4, (lower limb region)* | 67 | 4.79 | -27.86  37.43 | 0.77 | 0.94 |
|  | *A4ll, area 4, (lower limb region)* | 68 | -30.36 | -58.68  -2.04 | 0.04 | 0.46 |
| **Temp. Lobe** | *A38m, medial area 38* | 69 | 29.91 | 7.77  52.04 | 0.01 | 0.33 |
|  | *A38m, medial area 38* | 70 | 19.08 | -3.20  41.37 | 0.09 | 0.53 |
|  | *A41/42, area 41/42* | 71 | -15.66 | -46.10  14.79 | 0.31 | 0.76 |
|  | *A41/42, area 41/42* | 72 | -15.15 | -39.38  9.08 | 0.22 | 0.70 |
|  | *TE1.0 and TE1.2* | 73 | 4.16 | -33.78  42.10 | 0.83 | 0.96 |
|  | *TE1.0 and TE1.2* | 74 | 10.01 | -22.44  42.46 | 0.55 | 0.89 |
|  | *A22c, caudal area 22* | 75 | 7.16 | -18.39  32.72 | 0.58 | 0.87 |
|  | *A22c, caudal area 22* | 76 | 12.43 | -24.76  49.62 | 0.51 | 0.90 |
|  | *A38l, lateral area 38* | 77 | 26.16 | -2.89  55.21 | 0.08 | 0.56 |
|  | *A38l, lateral area 38* | 78 | 4.57 | -27.28  36.43 | 0.78 | 0.94 |
|  | *A22r, rostral area 22* | 79 | -14.79 | -44.63  15.05 | 0.33 | 0.78 |
|  | *A22r, rostral area 22* | 80 | -17.64 | -36.23  0.95 | 0.06 | 0.55 |
|  | *A21c, caudal area 21* | 81 | 12.51 | -11.46  36.48 | 0.31 | 0.75 |
|  | *A21c, caudal area 21* | 82 | 19.39 | -7.26  46.04 | 0.15 | 0.70 |
|  | *A21r, rostral area 21* | 83 | 18.55 | -16.29  53.39 | 0.30 | 0.75 |
|  | *A21r, rostral area 21* | 84 | 18.24 | -11.30  47.77 | 0.23 | 0.70 |
|  | *A37dl, dorsolateral area37* | 85 | 12.74 | -18.60  44.07 | 0.43 | 0.87 |
|  | *A37dl, dorsolateral area37* | 86 | 16.91 | -13.33  47.15 | 0.27 | 0.76 |
|  | *aSTS, anterior superior temporal sulcus* | 87 | -2.03 | -28.88  24.81 | 0.88 | 0.97 |
|  | *aSTS, anterior superior temporal sulcus* | 88 | 6.72 | -14.74  28.18 | 0.54 | 0.89 |
|  | *A20iv, intermediate ventral area 20* | 89 | -5.04 | -24.37  14.30 | 0.61 | 0.86 |
|  | *A20iv, intermediate ventral area 20* | 90 | -3.39 | -14.71  7.94 | 0.56 | 0.88 |
|  | *A37elv, extreme lateroventral area37* | 91 | 0.98 | -20.06  22.01 | 0.93 | 0.99 |
|  | *A37elv, extreme lateroventral area37* | 92 | 11.98 | -6.45  30.40 | 0.20 | 0.69 |
|  | *A20r, rostral area 20* | 93 | 46.62 | 15.86  77.39 | 0.00 | 0.24 |
|  | *A20r, rostral area 20* | 94 | 35.14 | 4.57  65.71 | 0.02 | 0.37 |
|  | *A20il, intermediate lateral area 20* | 95 | 28.07 | 1.28  54.86 | 0.04 | 0.49 |
|  | *A20il, intermediate lateral area 20* | 96 | 10.19 | -16.21  36.59 | 0.45 | 0.86 |
|  | *A37vl, ventrolateral area 37* | 97 | 40.97 | 6.49  75.46 | 0.02 | 0.35 |
|  | *A37vl, ventrolateral area 37* | 98 | 8.99 | -20.39  38.36 | 0.55 | 0.88 |
|  | *A20cl, caudolateral of area 20* | 99 | 17.17 | -13.58  47.92 | 0.27 | 0.76 |
|  | *A20cl, caudolateral of area 20* | 100 | -8.13 | -37.39  21.13 | 0.59 | 0.86 |
|  | *A20cv, caudoventral of area 20* | 101 | 39.00 | -0.78  78.78 | 0.05 | 0.56 |
|  | *A20cv, caudoventral of area 20* | 102 | 33.17 | -7.14  73.49 | 0.11 | 0.55 |
|  | *A20rv, rostroventral area 20* | 103 | 16.81 | -3.57  37.19 | 0.11 | 0.55 |
|  | *A20rv, rostroventral area 20* | 104 | 17.81 | -8.25  43.86 | 0.18 | 0.69 |
|  | *A37mv, medioventral area37* | 105 | -17.10 | -41.26  7.07 | 0.17 | 0.68 |
|  | *A37mv, medioventral area37* | 106 | 1.21 | -35.29  37.71 | 0.95 | 1.00 |
|  | *A37lv, lateroventral area37* | 107 | -3.87 | -21.32  13.57 | 0.66 | 0.88 |
|  | *A37lv, lateroventral area37* | 108 | -10.44 | -37.38  16.51 | 0.45 | 0.87 |
|  | *A35/36r, rostral area 35/36* | 109 | 2.53 | -1.53  6.59 | 0.22 | 0.70 |
|  | *A35/36r, rostral area 35/36* | 110 | 1.42 | -0.76  3.61 | 0.20 | 0.70 |
|  | *A35/36c, caudal area 35/36* | 111 | -0.29 | -1.96  1.39 | 0.74 | 0.93 |
|  | *A35/36c, caudal area 35/36* | 112 | 2.45 | -0.27  5.16 | 0.08 | 0.55 |
|  | *TL, area TL (lateral PPHC, posterior parahippocampal gyrus)* | 113 | 0.14 | -0.51  0.80 | 0.66 | 0.88 |
|  | *TL, area TL (lateral PPHC, posterior parahippocampal gyrus)* | 114 | 0.13 | -0.12  0.38 | 0.32 | 0.76 |
|  | *A28/34, area 28/34 (EC, entorhinal cortex)* | 115 | 1.02 | -1.19  3.24 | 0.37 | 0.82 |
|  | *A28/34, area 28/34 (EC, entorhinal cortex)* | 116 | -0.87 | -1.79  0.05 | 0.06 | 0.57 |
|  | *TI, area TI(temporal agranular insular cortex)* | 117 | 0.04 | -0.03  0.10 | 0.27 | 0.76 |
|  | *TI, area TI(temporal agranular insular cortex)* | 118 | 0.02 | -1.22  1.26 | 0.97 | 0.99 |
|  | *TH, area TH (medial PPHC)* | 119 | 1.65 | -0.69  3.99 | 0.17 | 0.66 |
|  | *TH, area TH (medial PPHC)* | 120 | 1.40 | -4.42  7.22 | 0.64 | 0.88 |
|  | *rpSTS, rostroposterior superior temporal sulcus* | 121 | 2.10 | -22.20  26.40 | 0.87 | 0.98 |
|  | *rpSTS, rostroposterior superior temporal sulcus* | 122 | 11.81 | -15.95  39.57 | 0.40 | 0.84 |
|  | *cpSTS, caudoposterior superior temporal sulcus* | 123 | -0.88 | -30.01  28.25 | 0.95 | 0.99 |
|  | *cpSTS, caudoposterior superior temporal sulcus* | 124 | 14.67 | -6.87  36.21 | 0.18 | 0.68 |
| **Parietal Lobe** | *A7r, rostral area 7* | 125 | -18.49 | -44.74  7.76 | 0.17 | 0.68 |
|  | *A7r, rostral area 7* | 126 | -2.51 | -31.59  26.58 | 0.87 | 0.97 |
|  | *A7c, caudal area 7* | 127 | -6.60 | -35.39  22.20 | 0.65 | 0.88 |
|  | *A7c, caudal area 7* | 128 | -17.47 | -43.28  8.33 | 0.18 | 0.68 |
|  | *A5l, lateral area 5* | 129 | -6.16 | -31.36  19.05 | 0.63 | 0.87 |
|  | *A5l, lateral area 5* | 130 | -6.14 | -28.11  15.83 | 0.58 | 0.87 |
|  | *A7pc, postcentral area 7* | 131 | -5.70 | -31.02  19.63 | 0.66 | 0.88 |
|  | *A7pc, postcentral area 7* | 132 | -23.91 | -50.76  2.94 | 0.08 | 0.52 |
|  | *A7ip, intraparietal area 7(hIP3)* | 133 | 4.97 | -20.06  30.00 | 0.70 | 0.90 |
|  | *A7ip, intraparietal area 7(hIP3)* | 134 | -10.00 | -35.89  15.88 | 0.45 | 0.86 |
|  | *A39c, caudal area 39(PGp)* | 135 | -15.58 | -54.47  23.31 | 0.43 | 0.88 |
|  | *A39c, caudal area 39(PGp)* | 136 | -9.83 | -34.43  14.77 | 0.43 | 0.87 |
|  | *A39rd, rostrodorsal area 39(Hip3)* | 137 | 2.54 | -25.99  31.07 | 0.86 | 0.98 |
|  | *A39rd, rostrodorsal area 39(Hip3)* | 138 | -0.58 | -21.50  20.34 | 0.96 | 0.98 |
|  | *A40rd, rostrodorsal area 40(PFt)* | 139 | -4.00 | -23.47  15.47 | 0.69 | 0.90 |
|  | *A40rd, rostrodorsal area 40(PFt)* | 140 | -19.53 | -39.86  0.80 | 0.06 | 0.57 |
|  | *A40c, caudal area 40(PFm)* | 141 | 11.11 | -13.29  35.50 | 0.37 | 0.82 |
|  | *A40c, caudal area 40(PFm)* | 142 | -25.03 | -54.51  4.45 | 0.10 | 0.53 |
|  | *A39rv, rostroventral area 39(PGa)* | 143 | -20.35 | -35.55  -5.15 | 0.01 | 0.27 |
|  | *A39rv, rostroventral area 39(PGa)* | 144 | -5.97 | -24.83  12.88 | 0.53 | 0.91 |
|  | *A40rv, rostroventral area 40(PFop)* | 145 | -7.91 | -35.01  19.20 | 0.57 | 0.88 |
|  | *A40rv, rostroventral area 40(PFop)* | 146 | -6.06 | -24.04  11.93 | 0.51 | 0.91 |
|  | *A7m, medial area 7(PEp)* | 147 | -11.78 | -38.98  15.43 | 0.40 | 0.83 |
|  | *A7m, medial area 7(PEp)* | 148 | -2.57 | -28.37  23.23 | 0.85 | 0.96 |
|  | *A5m, medial area 5(PEm)* | 149 | -4.62 | -33.95  24.71 | 0.76 | 0.94 |
|  | *A5m, medial area 5(PEm)* | 150 | 21.57 | -3.82  46.95 | 0.10 | 0.54 |
|  | *dmPOS, dorsomedial parietooccipital sulcus(PEr)* | 151 | -19.62 | -39.50  0.26 | 0.05 | 0.57 |
|  | *dmPOS, dorsomedial parietooccipital sulcus(PEr)* | 152 | -9.38 | -27.14  8.39 | 0.30 | 0.76 |
|  | *A31, area 31 (Lc1)* | 153 | -4.01 | -33.21  25.19 | 0.79 | 0.94 |
|  | *A31, area 31 (Lc1)* | 154 | 10.52 | -16.09  37.12 | 0.44 | 0.88 |
|  | *A1/2/3ulhf, area 1/2/3(upper limb, head and face region)* | 155 | -20.46 | -42.59  1.67 | 0.07 | 0.56 |
|  | *A1/2/3ulhf, area 1/2/3(upper limb, head and face region)* | 156 | -5.67 | -26.11  14.77 | 0.59 | 0.85 |
|  | *A1/2/3tonIa, area 1/2/3(tongue and larynx region)* | 157 | 9.18 | -20.82  39.18 | 0.55 | 0.89 |
|  | *A1/2/3tonIa, area 1/2/3(tongue and larynx region)* | 158 | 11.20 | -16.23  38.63 | 0.42 | 0.88 |
|  | *A2, area 2* | 159 | -3.61 | -27.18  19.97 | 0.76 | 0.94 |
|  | *A2, area 2* | 160 | -8.25 | -30.25  13.74 | 0.46 | 0.87 |
|  | *A1/2/3tru, area1/2/3(trunk region)* | 161 | 7.06 | -21.11  35.23 | 0.62 | 0.87 |
|  | *A1/2/3tru, area1/2/3(trunk region)* | 162 | -13.73 | -36.32  8.85 | 0.23 | 0.70 |
| **Insular Lobe** | *G, hypergranular insula* | 163 | 15.91 | -4.87  36.70 | 0.13 | 0.63 |
|  | *G, hypergranular insula* | 164 | 16.77 | -2.56  36.11 | 0.09 | 0.52 |
|  | *vIa, ventral agranular insula* | 165 | 0.21 | -4.46  4.89 | 0.93 | 0.99 |
|  | *vIa, ventral agranular insula* | 166 | 2.62 | -3.16  8.40 | 0.37 | 0.81 |
|  | *dIa, dorsal agranular insula* | 167 | 7.82 | -3.50  19.15 | 0.18 | 0.69 |
|  | *dIa, dorsal agranular insula* | 168 | 1.86 | -8.42  12.13 | 0.72 | 0.93 |
|  | *vId/vIg, ventral dysgranular and granular insula* | 169 | 13.54 | -1.76  28.84 | 0.08 | 0.51 |
|  | *vId/vIg, ventral dysgranular and granular insula* | 170 | 12.99 | -4.34  30.33 | 0.14 | 0.66 |
|  | *dIg, dorsal granular insula* | 171 | -4.21 | -24.95  16.52 | 0.69 | 0.89 |
|  | *dIg, dorsal granular insula* | 172 | 4.06 | -13.42  21.54 | 0.65 | 0.88 |
|  | *dId, dorsal dysgranular insula* | 173 | -2.42 | -17.17  12.33 | 0.75 | 0.94 |
|  | *dId, dorsal dysgranular insula* | 174 | -8.78 | -25.04  7.48 | 0.29 | 0.76 |
| **Limbic Lobe** | *A23d, dorsal area 23* | 175 | 9.71 | -8.49  27.90 | 0.30 | 0.76 |
|  | *A23d, dorsal area 23* | 176 | 10.24 | -5.71  26.19 | 0.21 | 0.68 |
|  | *A24rv, rostroventral area 24* | 177 | -4.93 | -9.05  -0.81 | 0.02 | 0.36 |
|  | *A24rv, rostroventral area 24* | 178 | 1.38 | -9.39  12.15 | 0.80 | 0.95 |
|  | *A32p, pregenual area 32* | 179 | 10.36 | -19.89  40.60 | 0.50 | 0.90 |
|  | *A32p, pregenual area 32* | 180 | 15.79 | -3.13  34.72 | 0.10 | 0.55 |
|  | *A23v, ventral area 23* | 181 | -9.82 | -38.28  18.65 | 0.50 | 0.90 |
|  | *A23v, ventral area 23* | 182 | 26.87 | -1.95  55.68 | 0.07 | 0.57 |
|  | *A24cd, caudodorsal area 24* | 183 | 18.36 | -2.17  38.90 | 0.08 | 0.54 |
|  | *A24cd, caudodorsal area 24* | 184 | 11.19 | -6.09  28.48 | 0.20 | 0.69 |
|  | *A23c, caudal area 23* | 185 | -12.77 | -37.81  12.27 | 0.32 | 0.76 |
|  | *A23c, caudal area 23* | 186 | 11.84 | -13.66  37.34 | 0.36 | 0.82 |
|  | *A32sg, subgenual area 32* | 187 | 10.14 | -21.09  41.37 | 0.52 | 0.90 |
|  | *A32sg, subgenual area 32* | 188 | 18.64 | 0.21  37.07 | 0.05 | 0.56 |
| **Occ.**  **Lobe** | *cLinG, caudal lingual gyrus* | 189 | -10.08 | -46.40  26.23 | 0.59 | 0.86 |
|  | *cLinG, caudal lingual gyrus* | 190 | -64.97 | -106.67  -23.28 | 0.00 | 0.28 |
|  | *rCunG, rostral cuneus gyrus* | 191 | -15.81 | -42.65  11.03 | 0.25 | 0.74 |
|  | *rCunG, rostral cuneus gyrus* | 192 | -8.22 | -36.90  20.46 | 0.57 | 0.88 |
|  | *cCunG, caudal cuneus gyrus* | 193 | 6.42 | -33.17  46.00 | 0.75 | 0.94 |
|  | *cCunG, caudal cuneus gyrus* | 194 | -0.58 | -31.07  29.91 | 0.97 | 0.99 |
|  | *rLinG, rostral lingual gyrus* | 195 | -6.99 | -31.89  17.90 | 0.58 | 0.88 |
|  | *rLinG, rostral lingual gyrus* | 196 | -33.64 | -58.89  -8.39 | 0.01 | 0.25 |
|  | *vmPOS,ventromedial parietooccipital sulcus* | 197 | -22.34 | -41.83  -2.85 | 0.02 | 0.36 |
|  | *vmPOS,ventromedial parietooccipital sulcus* | 198 | -5.12 | -23.24  13.00 | 0.58 | 0.88 |
|  | *mOccG, middle occipital gyrus* | 199 | 2.86 | -41.80  47.53 | 0.90 | 0.97 |
|  | *mOccG, middle occipital gyrus* | 200 | -1.05 | -40.86  38.75 | 0.96 | 0.98 |
|  | *V5/MT+, area V5/MT+* | 201 | 0.08 | -36.75  36.91 | 1.00 | 1.00 |
|  | *V5/MT+, area V5/MT+* | 202 | -21.60 | -59.68  16.47 | 0.27 | 0.76 |
|  | *OPC, occipital polar cortex* | 203 | -0.92 | -28.63  26.80 | 0.95 | 1.00 |
|  | *OPC, occipital polar cortex* | 204 | -38.27 | -68.23  -8.31 | 0.01 | 0.30 |
|  | *iOccG, inferior occipital gyrus* | 205 | 4.97 | -30.20  40.15 | 0.78 | 0.93 |
|  | *iOccG, inferior occipital gyrus* | 206 | 11.87 | -21.80  45.53 | 0.49 | 0.90 |
|  | *msOccG, medial superior occipital gyrus* | 207 | -2.86 | -36.94  31.22 | 0.87 | 0.97 |
|  | *msOccG, medial superior occipital gyrus* | 208 | 4.87 | -28.25  37.98 | 0.77 | 0.94 |
|  | *lsOccG, lateral superior occipital gyrus* | 209 | -2.50 | -27.39  22.38 | 0.84 | 0.97 |
|  | *lsOccG, lateral superior occipital gyrus* | 210 | -14.19 | -41.09  12.70 | 0.30 | 0.75 |
| **Deep Gray Matter** | *mAmyg, medial amygdala* | 211 | 0.09 | 0.02  0.16 | 0.01 | 0.30 |
|  | *mAmyg, medial amygdala* | 212 | 0.01 | -0.15  0.18 | 0.87 | 0.97 |
|  | *lAmyg, lateral amygdala* | 213 | -0.00007 | -0.0003  0.0002 | 0.55 | 0.88 |
|  | *lAmyg, lateral amygdala* | 214 | -0.01 | -0.05  0.02 | 0.52 | 0.90 |
|  | *rHipp, rostral hippocampus* | 215 | -0.05 | -1.78  1.67 | 0.95 | 1.00 |
|  | *rHipp, rostral hippocampus* | 216 | 2.09 | -3.23  7.40 | 0.44 | 0.87 |
|  | *cHipp, caudal hippocampus* | 217 | -4.99 | -13.95  3.96 | 0.27 | 0.75 |
|  | *cHipp, caudal hippocampus* | 218 | 2.27 | -6.21  10.74 | 0.60 | 0.87 |
|  | *vCa, ventral caudate* | 219 | -0.86 | -3.60  1.88 | 0.54 | 0.91 |
|  | *vCa, ventral caudate* | 220 | -0.34 | -1.38  0.69 | 0.51 | 0.90 |
|  | *GP, globus pallidus* | 221 | 0.00 | -0.01  0.00 | 0.21 | 0.67 |
|  | *GP, globus pallidus* | 222 | -0.0003 | -0.0007  0.0001 | 0.18 | 0.69 |
|  | *NAC, nucleus accumbens* | 223 | -0.66 | -1.88  0.56 | 0.29 | 0.76 |
|  | *NAC, nucleus accumbens* | 224 | -4.13 | -10.47  2.20 | 0.20 | 0.72 |
|  | ***vmPu, ventromedial putamen*** | **225** | **-0.03** | **-0.04**  **-0.01** | **<0.0001** | **0.01** |
|  | *vmPu, ventromedial putamen* | 226 | -0.00009 | -0.000001  0.000000 | 0.87 | 0.97 |
|  | *dCa, dorsal caudate* | 227 | -0.48 | -10.35  9.40 | 0.92 | 0.99 |
|  | *dCa, dorsal caudate* | 228 | -1.99 | -6.05  2.07 | 0.34 | 0.78 |
|  | *dlPu, dorsolateral putamen* | 229 | -0.01 | -0.03  0.01 | 0.51 | 0.90 |
|  | *dlPu, dorsolateral putamen* | 230 | -0.01 | -0.01  0.00 | 0.21 | 0.69 |
|  | *mPFtha, medial pre-frontal thalamus* | 231 | -0.01 | -0.09  0.07 | 0.82 | 0.95 |
|  | *mPFtha, medial pre-frontal thalamus* | 232 | 0.06 | -0.10  0.22 | 0.44 | 0.88 |
|  | *mPMtha, pre-motor thalamus* | 233 | -0.0000002 | -0.000001  0.000000 | 0.22 | 0.69 |
|  | *mPMtha, pre-motor thalamus* | 234 | -0.0000148 | -0.0001  0.0001 | 0.82 | 0.96 |
|  | *Stha, sensory thalamus* | 235 | -0.0000004 | -0.000002  0.000001 | 0.54 | 0.89 |
|  | *Stha, sensory thalamus* | 236 | 0.0000010 | -0.000004  0.000006 | 0.69 | 0.89 |
|  | *rTtha, rostral temporal thalamus* | 237 | -2.12 | -4.29  0.05 | 0.06 | 0.55 |
|  | *rTtha, rostral temporal thalamus* | 238 | -6.32 | -11.43  -1.21 | 0.02 | 0.34 |
|  | *PPtha, posterior parietal thalamus* | 239 | -0.0004 | -0.0001  0.0001 | 0.36 | 0.82 |
|  | *PPtha, posterior parietal thalamus* | 240 | -0.04 | -0.17  0.09 | 0.54 | 0.89 |
|  | *Otha, occipital thalamus* | 241 | 0.34 | -0.99  1.67 | 0.62 | 0.87 |
|  | *Otha, occipital thalamus* | 242 | 1.75 | -0.21  3.72 | 0.08 | 0.54 |
|  | *cTtha, caudal temporal thalamus* | 243 | -0.50 | -2.25  1.25 | 0.57 | 0.88 |
|  | *cTtha, caudal temporal thalamus* | 244 | -0.01 | -1.41  1.40 | 0.99 | 1.00 |
|  | *lPFtha, lateral pre-frontal thalamus* | 245 | -0.00019 | -0.0008  0.0004 | 0.54 | 0.90 |
|  | *lPFtha, lateral pre-frontal thalamus* | 246 | -0.00012 | -0.0003  0.00002 | 0.08 | 0.50 |

Abbreviations: CI: confidence interval; Occ.: occipital; Temp: temporal

^a^ Results from multilevel mixed-effects models determining the effect of group status, age, sex, visit (expressed as months from baseline), and group-visit interaction on eigenvector centrality over time.

^b^ Benjamini and Hochberg false discovery rate correction for multiple comparisons. Q-value= (p-value*N tests)/test rank.
